# Electrophoretic Deposition of Chitosan Coatings on the Porous Titanium Substrate

**DOI:** 10.3390/jfb15070190

**Published:** 2024-07-09

**Authors:** Julia Flesińska, Magdalena Szklarska, Izabela Matuła, Adrian Barylski, Sylwia Golba, Julia Zając, Maciej Gawlikowski, Przemysław Kurtyka, Barbara Ilnicka, Grzegorz Dercz

**Affiliations:** 1 Institute of Materials Engineering, University of Silesia in Katowice, 75 Pułku Piechoty St. 1 A, 41-500 Chorzów, Poland; julia.flesinska@op.pl (J.F.); izabela.matula@us.edu.pl (I.M.); adrian.barylski@us.edu.pl (A.B.); sylwia.golba@us.edu.pl (S.G.); jzajac2000@gmail.com (J.Z.); 2 Foundation of Cardiac Surgery Development, Institute of Heart Prostheses, 35a Wolności St., 41-800 Zabrze, Poland; mgawlik@frk.pl (M.G.); pkurtyka@frk.pl (P.K.); 3 Faculty of Biomedical Engineering, Silesian University of Technology, Roosevelt’s Str. 40, 41-800 Zabrze, Poland; 4 Faculty of Automatic Control, Electronics and Computer Science, Silesian University of Technology, Akademicka 16 St., 44-100 Gliwice, Poland; barbiln872@student.polsl.pl

**Keywords:** porous titanium, chitosan, electrophoretic deposition

## Abstract

Medicine is looking for solutions to help implant patients recover more smoothly. The porous implants promote osteointegration, thereby providing better stabilization. Introducing porosity into metallic implants enhances their biocompatibility and facilitates osteointegration. The introduction of porosity is also associated with a reduction in Young’s modulus, which reduces the risk of tissue outgrowth around the implant. However, the risk of chronic inflammation remains a concern, necessitating the development of coatings to mitigate adverse reactions. An interesting biomaterial for such modifications is chitosan, which has antimicrobial, antifungal, and osteointegration properties. In the present work, a porous titanium biomaterial was obtained by powder metallurgy, and electrophoretic deposition of chitosan coatings was used to modify its surface. This study investigated the influence of ethanol content in the deposition solution on the quality of chitosan coatings. The EPD process facilitates the control of coating thickness and morphology, with higher voltages resulting in thicker coatings and increased pore formation. Ethanol concentration in the solution affects coating quality, with higher concentrations leading to cracking and peeling. Optimal coating conditions (30 min/10 V) yield high-quality coatings, demonstrating excellent cell viability and negligible cytotoxicity. The GIXD and ATR-FTIR analysis confirmed the presence of deposited chitosan coatings on Ti substrates. The microstructure of the chitosan coatings was examined by scanning electron microscopy. Biological tests showed no cytotoxicity of the obtained materials, which allows for further research and the possibility of their use in medicine. In conclusion, EPD offers a viable method for producing chitosan-based coatings with controlled properties for biomedical applications, ensuring enhanced patient outcomes and implant performance.

## 1. Introduction

Biomaterials have been an important part of medicine for years. Metal-based biomaterials are very popular due to their unique properties [1]. Metallic biomaterials are also eagerly used in medicine due to the possibility of modifying their properties and surfaces [2]. This enables the continuous development of implantology and improves the quality and comfort of a patient’s life undergoing implant procedures. Metallic implants are used mainly in bone surgery [3]. Introducing porosity into the metallic implant reduces Young’s modulus and brings its value closer to the typical bone. This reduces the risk of tissue stiffness and resorption around the implant. In addition, the presence of pores on the surface promotes the ingrowth of bone tissue into the implant and its permanent connection to the bone, which results in the process of osteointegration and healing being more efficient [4,5]. In the case of dental implants, this is especially important for patient comfort. It should be noted that the placement of dental implants carries the risk of developing chronic inflammation. The risk stems from the relatively easy penetration of bacteria into the peri-implant tissues caused by inadequate oral hygiene [6]. A solution to the problem of bone tissue integration being disrupted with the implant and the possibility of corrosion development and acute inflammation is covering the implant surface with biopolymer coatings [7].

Such coating could also act as a carrier for analgesics or anti-inflammatory medications. An attractive natural polymer is chitosan, distinguished by its antibacterial, antifungal, and antiviral properties. These characteristics are unparalleled among other medical biopolymers, such as alginate or hyaluronate [8,9]. Chitosan is a biomaterial; therefore, it meets the basic requirements of biocompatibility and non-toxicity and does not cause harmful or undesirable reactions in the body [10,11,12,13]. Chitosan shows biodegradability and accelerates the process of wound healing due to its hemostatic properties, bioactivity, and cell proliferation. It is worth mentioning that this material is susceptible to all kinds of structural modifications, so new derivatives with different properties can be created [14]. In addition, chitosan has better solubility than chitin, which is particularly desirable in the process of applying coatings [15]. In vitro studies have also shown that chitosan is more cytocompatible than chitin [16]. Chitosan is a biopolymer with the necessary qualities to be used in medicine and unique qualities that can realistically improve patients’ health.

Among the array of techniques utilized for applying biopolymer coatings onto metallic substrates, such as dip coating or sol-gel methods, one prominent method stands out: the electrophoretic deposition (EPD) technique. This versatile technique allows coatings to be applied to all sorts of complex product surfaces. In addition, the surface of large parts with any irregularities, pores, or roughness can be modified using this method [17]. It is faster than dip coating or sol-gel methods and allows for the morphology of the coatings to be tailored with process parameter changes. A significant advantage of the EPD process is the simplicity of its execution and the low cost associated with the required equipment, unlike chemical vapor deposition (CVD) and physical vapor deposition (PVD), which require expensive and complex equipment [18]. Additionally, CVD often requires high temperatures, which may not be suitable for all materials. The low cost associated with the required tooling can lead to increased production inputs and product diffusion [19]. The electrophoretic deposition of coatings is carried out with a direct current. Particles of a determined charge in the suspension move towards the counter-electrode and form a deposit on its conductive surface. Under the influence of an electric field, the polymer macromolecules or particles presented in the solution migrate towards the electrode, where the coagulation process occurs, and the coatings are formed [20]. EPD is a process tightly controlled by parameters such as time and voltage and the deposition solution [21]. Modifying the parameters makes it possible to influence the thickness and morphology of the resulting coatings. Many types of coatings can be obtained using this method, including gradient, multilayer, and homogeneous coatings. The coatings produced are also environmentally safe [19]. The great advantage of the EPD method, which distinguishes it from other coating deposition methods, especially those for medical applications, is that the process can be carried out at low temperatures. The ability to carry out the process at low temperatures is particularly important when the coatings produced act as controlled drug delivery since pharmaceuticals are sensitive to high temperatures [22].

Due to its nature, chitosan dissolves in acidic solutions such as acetic acid, malonic acid, or citric acid, where it undergoes protonation and acquires a positive charge. The literature shows that increasing the deposition voltage in the EPD process increases the amount of deposited polymer. However, this also raises the likelihood of obtaining a porous coating due to the rapid release of gases at the electrodes [23]. Studies by Mahmoodi et al., Cordero-Arias et al., or Błoniarz et al. indicate that organic liquids, especially alcohols, can mitigate the adverse effects of aqueous solutions on gas generation at the cathode and anode surfaces, leading to coatings with fewer pores and thus better adhesion to the substrate. Furthermore, various particles can be deposited in the EPD process along with chitosan, which generally disperse better in ethanol than in water. Therefore, this study investigated the impact of ethanol content in the deposition solution on the quality of the obtained coatings [23,24,25,26].

## 2. Materials and Methods

### 2.1. Material Preparation

The substrate of the samples was produced from titanium (Atlantic Equipment Engineers (AEE), New York, NY, USA; purity 99.5%,) which was agglomerated in a planetary ball mill (process parameters: 200 rpm for 10 h). The cylindrical samples were pressed by isostatic uniaxial cold pressing (FACOM W.415WBA Stanley Black & Decker, Limonest, France) at 250 MPa. They were then sintered in a furnace for 24 h at 1000 °C and cooled with the furnace. The samples were cut into discs 3 mm high and mechanically polished using a sequence of abrasive papers with increasing grit sizes, specifically #600, #800, #1200, and #2000 gradation, until a smooth surface finish was obtained. The samples were cleaned in an ultrasonic cleaner using distilled water and ethanol. The samples prepared in this way served as the base material for the electrophoretic deposition process of chitosan coatings.

A commercial chitosan powder (Sigma-Aldrich, Steinheim, Germany) with an average molecular weight of 80 kDa and a 75–85% deacetylation degree, citric acid (Acros Organic, Geel, Antwerpen, Belgium; 99.6%), distilled water and ethyl alcohol (anhydrous 99.8%) were used to prepare solutions for electrophoretic deposition. The electrophoretic coating deposition process was carried out with four different solutions:

- starting solution: 2% citric acid solution containing 1 g/dm^3^ of chitosan,
- solution 1: 2% citric acid solution in 25% ethanol containing 1 g/dm^3^ of chitosan,
- solution 2: 2% citric acid solution in 50% ethanol containing 1 g/dm^3^ of chitosan,
- solution 3: 2% citric acid solution in 75% ethanol containing 1 g/dm^3^ of chitosan

At deposition voltage of U = 2.5–20 V for time of deposition of t = 2.5–30 min at 25 °C. An electrochemical cell with two parallel electrodes spaced 1.5 cm apart was utilized for the EPD process. The titanium sample served as the working electrode, while a platinum foil acted as the counter electrode. Chitosan coatings were deposited using a KIKUSUI PWR800H high-current power supply (KIKUSUI, Yokohama, Japan). After deposition, the chitosan coatings were thoroughly rinsed with distilled water and dried at room temperature for 24 h.

### 2.2. Material Characterization

The X-ray diffraction (XRD) measurements were performed on the X’Pert Philips PW 3040/60 diffractometer operating (Panalytical B.V., Almelo, Holland) at I = 30 mA and U = 40 kV. The wavelength of radiation (λCu Kα) was 1.54178 Å. The grazing incidence X-ray diffraction (GIXD) patterns were registered in the 2θ range from 5 to 100° 2θ with step scanning at 0.05° 2θ steps and the angle of incidence α = 0.50°. Qualitative phase analysis was performed using the ICDD PDF 5+ database.

Attenuated total reflectance Fourier transform infrared (ATR-FTIR) spectroscopy (Shimadzu IR Prestige-21, Kyoto, Japan) was employed to identify the functional groups present in the produced chitosan coatings.

The microstructure of the Ti substrate and chitosan coatings was examined using an IX81 inverted Olympus microscope (Olympus, Tokyo, Japan) and a JEOL JSM-6480 scanning electron microscope (JEOL Ltd., Tokyo, Japan) (SEM), which was equipped with an energy-dispersive X-ray spectroscopy (EDS) IXRF detector (IXRF, Austin, TX, USA). 

The surface roughness of the examined biomaterials was determined using a Surftest SJ-500/P profilometer (Mitutoyo, Kawasaki, Japan). Surface profile variations were measured with a step size of 0.1 μm and a speed of 200 μm/s over a length of approximately 7 mm. The recorded parameters were processed and analyzed using the FORMTRACEPAK 5.303 software.

Micromechanical testing (microindentation) was conducted using a Micro Combi Tester—MCT3 (Anton Paar, Corcelles-Cormondrèche, Switzerland). A Vickers diamond indenter (V-M 86) was used with a maximum load of 100 mN. Loading and unloading were performed over a period of 30 s (at a rate of 200 mN/min), with a dwell time at maximum load of 10 s. Seven indents were made for each sample. Measurements were performed in accordance with the ISO 14577 standard [27]. Hardness H_IT_ and elastic modulus E_IT_ were determined using the Oliver–Pharr method [28]. 

The cytotoxicity of the obtained biomaterials was evaluated through indirect contact tests. Before testing, the samples were sterilized with ethylene oxide. The samples were placed in the extraction medium, which was a complete culture medium. 10 mL of extraction medium was used for 1 g of sample. Extraction was performed under constant shaking at 37 °C for 24 h. Cytotoxicity tests were performed based on the ISO 10993-5 standard [29]. The fibroblast line, clone L 929—American Type Culture Collection (ATCC), was used in biological studies, sixth passage, viability 94.65%. 250,000 living cells were seeded into 25 cm^2^ dishes, and 100,000 cells were seeded into the wells of 6-well plates. Cells were cultured for 24 h in Medium 199 supplemented with 10% FBS under standard conditions. Fresh medium, supplemented with extraction medium by adding 1 mL of extraction medium to 9 mL of culture medium, was administered to all cultures after incubation. Incubation of cells in such conditions lasted 24 h. In the study assaying metabolic activity, each set of 5 repetitions with cells in 6-well plates included one control without cells. To each well containing 1 mL of culture medium, 200 μL of sterile resazurin solution was added in PBS at a concentration of 0.15 mg/mL. These prepared plates were then incubated for 2 h under conditions identical to the culture conditions. After incubation, the absorbance of the culture medium was measured at a wavelength of 605 nm using an Infinite M Nano (Tecan) and the Magellan 7.2 software (Tecan Group Ltd., Männedorf, Switzerland). Complete reduction of resazurin to resorufin was achieved by autoclaving a portion of the culture medium with resazurin solution. The absorbance of this solution was treated as the background absorbance. This background absorbance was subtracted from the absorbance measured for each sample. The absorbance value for each repetition was subtracted from the value obtained for the corresponding control without cells, and the resulting value was then divided by the absorbance value of the control without cells. This determined the proportion of resazurin added to the culture converted to the product. A higher percentage indicated higher metabolic activity and, consequently, a greater number of cells.

## 3. Results and Discussion

### 3.1. Porous Ti Substrate Characterization

**Figure 1:**
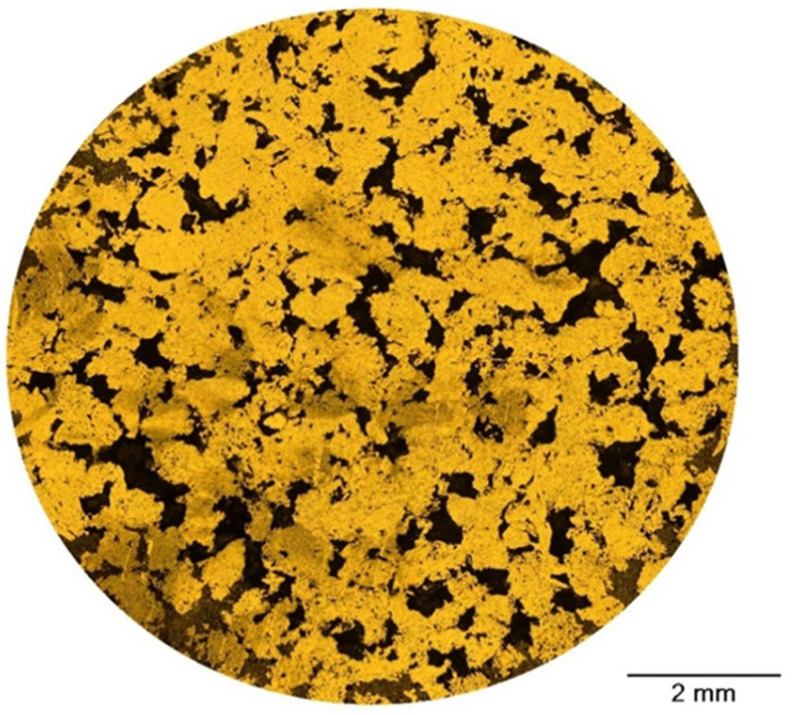
Microscopic image of the studied Ti sample surface.

The stereological analysis of the pore structures of the Ti substrate (Figure 1) shows that the relative porosity of the obtained material is 14%. Further investigation reveals that the average Feret’s diameter is 315 μm, and the circularity coefficient of pores is 0.238.

### 3.2. Chitosan Coatings Characterization

**Figure 2:**
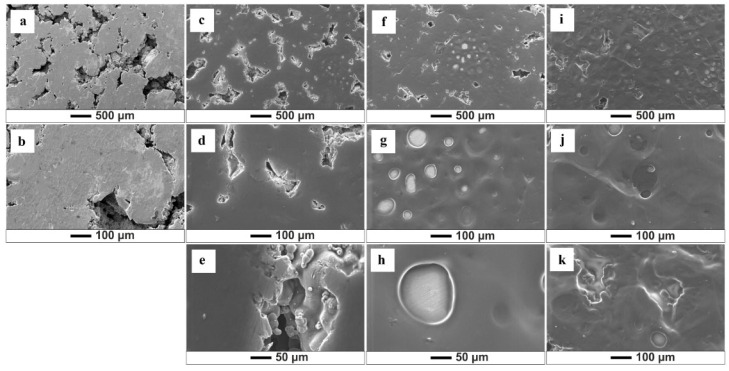
SEM images of the chitosan coatings deposited from the starting solution: (**a**,**b**) (5 min/2.5 V); (**c**–**e**) (5 min/10 V); (**f**–**h**) (5 min/20 V); (**i**–**k**) (30 min/10 V).

**Figure 3:**
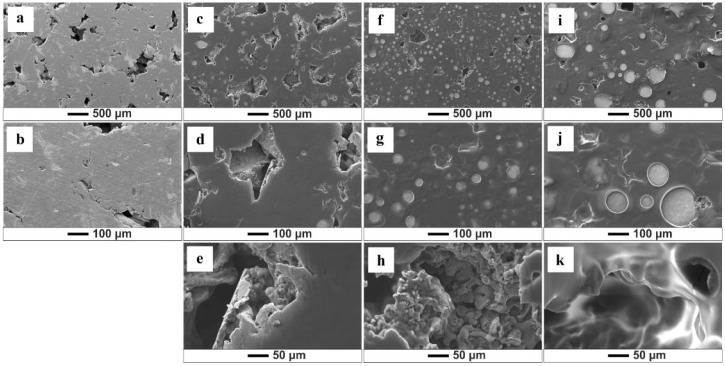
SEM images of the chitosan coatings deposited from solution 1 (25% ethanol): (**a**,**b**) (5 min/2.5 V); (**c**–**e**) (5 min/10 V); (**f**–**h**) (5 min/20 V); (**i**–**k**) (30 min/10 V).

**Figure 4:**
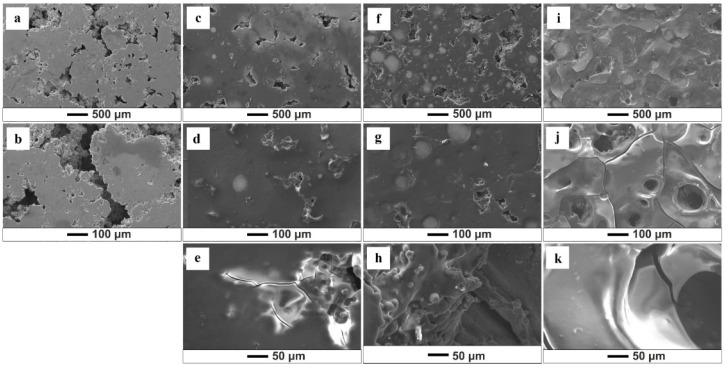
SEM images of the chitosan coatings deposited from solution 2 (50% ethanol): (**a**,**b**) (5 min/2.5 V); (**c**–**e**) (5 min/10 V); (**f**–**h**) (5 min/20 V); (**i**–**k**) (30 min/10 V).

**Figure 5:**
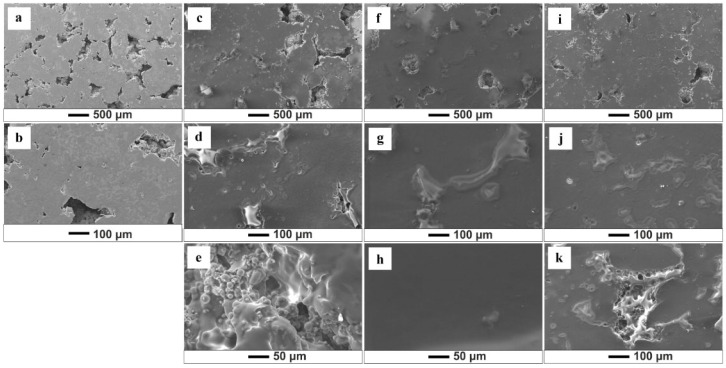
SEM images of the chitosan coatings deposited from solution 3 (75% ethanol): (**a**,**b**) (5 min/2.5 V); (**c**–**e**) (5 min/10 V); (**f**–**h**) (5 min/20 V); (**i**–**k**) (30 min/10 V).

SEM analysis allows the assessment of the quality of the obtained biopolymer coatings and shows that variations in deposition parameters and solutions influence the morphology of the formed coatings (Figure 2, Figure 3, Figure 4 and Figure 5). The results show that an increase in time and voltage positively affects the thickness and quality of the formed coatings. Additionally, with increasing the deposition voltage, the pores in chitosan coatings start to appear, leading to discontinuity of the coating where the pores occur. This phenomenon correlates with the kinetics of coating deposition and the magnitude of hydrogen evolution at the cathode stemming from the electrolytic decomposition of water. Increasing the coating deposition time to 30 min and decreasing the applied voltage to 10 V manifested less rapid hydrogen evolution, which decreased the number of pores in the obtained biopolymer coatings. The thickest and homogeneously distributed coating was formed by subjecting the samples to deposition for 30 min at 10 V, utilizing both the starting solution and solution 1. It should be noted that at the highest set parameters, i.e., 30 min/10 V, the chitosan coating covered the pores in the titanium substrate. The chitosan layer did not cover these pores at lower coating deposition parameters (5 min/10 V). SEM images also indicate a negative impact of the ethanol content in the solution on the quality and thickness of the obtained coatings. Figure 4 and Figure 5 show that increased ethanol content results in a cracked and peeling coating. Comparing the images of the coatings deposited from solution 3 relative to the coatings applied in the previous solution, one can see a significant change in the morphology of the sample surface (Figure 5). The bio-coatings deposited from this solution are thinner. Moreover, the coatings are homogeneous without visible pores, indicating that no hydrogen was released during the EPD process. The deposition of such thin coatings may be due to the high concentration of ethanol in the solution, which may have affected the solubility of chitosan in the solution and thus, adversely affected the EPD process. 

**Figure 6:**
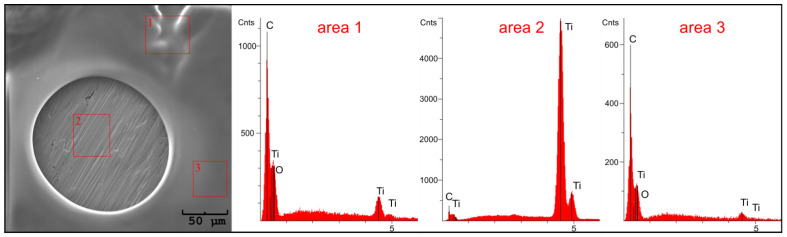
SEM images of the chitosan coatings deposited from the starting solution at 5 min/20 V, and the EDS analysis of the marked areas.

EDS analysis of selected fragments of the test sample revealed the presence of chitosan coatings on the titanium substrate in marked areas 1 and 3 (Figure 6). In these areas, there is a strong signal attributed to carbon. In the pores in area 2, only a strong signal from the Ti substrate is visible, indicating a coating discontinuity.

**Figure 7:**
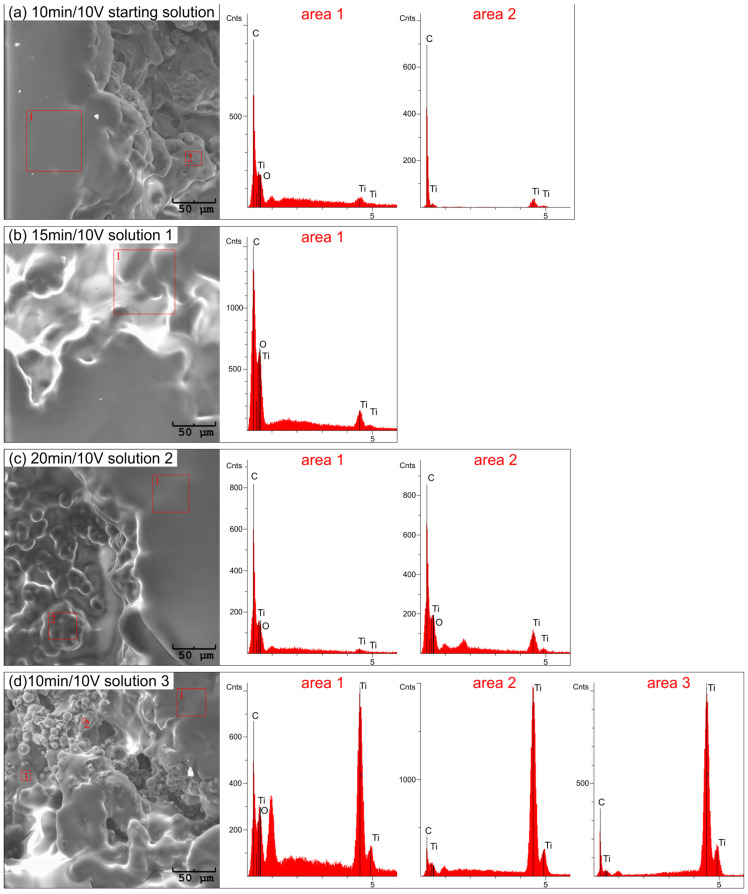
Result of EDS analysis of chitosan coatings on Ti substrate obtained from: (**a**) starting solution at 10 min/10 V, (**b**) solution 1 at 15 min/10 V, (**c**) solution 2 at 20 min/10 V, and (**d**) solution 3 at 10 min/10 V.

The EDS analysis also confirmed the presence of chitosan coatings in the pores of the titanium substrate (Figure 7). In selected samples, we observe complete coverage for the starting solution, solution 1, and solution 2 at deposition parameters of 10 min/10 V, 15 min/10 V, and 20 min/10 V, respectively (Figure 7a–c). Here, we detect a very weak signal from the Ti substrate and a strong signal from carbon, which is the main component of the coating. For solution 3 (Figure 7d), we observe partial coverage of the pore interior with the chitosan coating. A distinct peak from the Ti substrate and a weak signal from carbon is visible, which may indicate the presence of a thin biopolymer coating.

**Figure 8:**
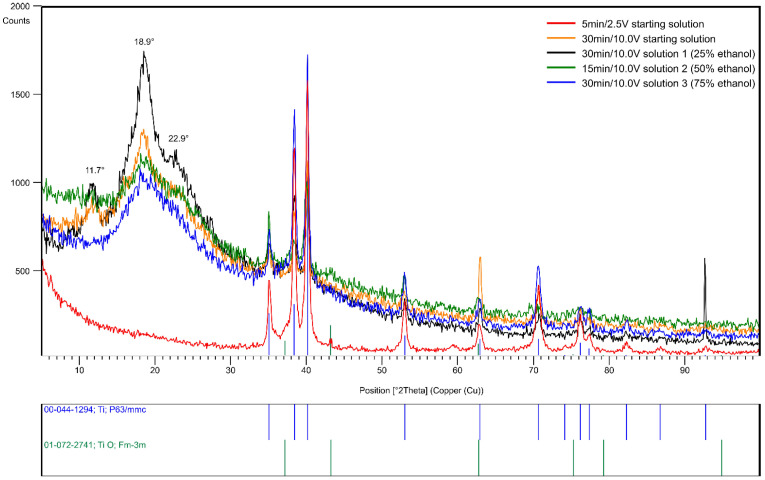
GIXD patterns for the angle of incidence α = 0.50° of chitosan coatings on Ti substrate.

The X-ray phase analysis identified two inorganic phases: α-Ti (ICDD PDF 00-044-1294) with space group P6_3_/mmc, and titanium oxide TiO with space group $\mathrm{Fm}\bar{3}m$ (Figure 8). In addition, the presence of organic chitosan is evidenced by the characteristic reflections, visible at 2θ = 11.7°, 18.9°, and 22.9° [30,31]. Examination of the XRD pattern suggests variations in the thickness of the chitosan coatings on the titanium substrate, influenced by the EPD parameters. Based on Figure 8, the sample deposited for 30 min at 10 V from solution 1 with 25% ethanol has the highest thickness. The XRD pattern of the sample deposited from the starting solution at 2.5 V for 5 min does not show reflection from chitosan. This may indicate the absence of a chitosan coating or the formed coating is too thin to be detected. 

**Figure 9:**
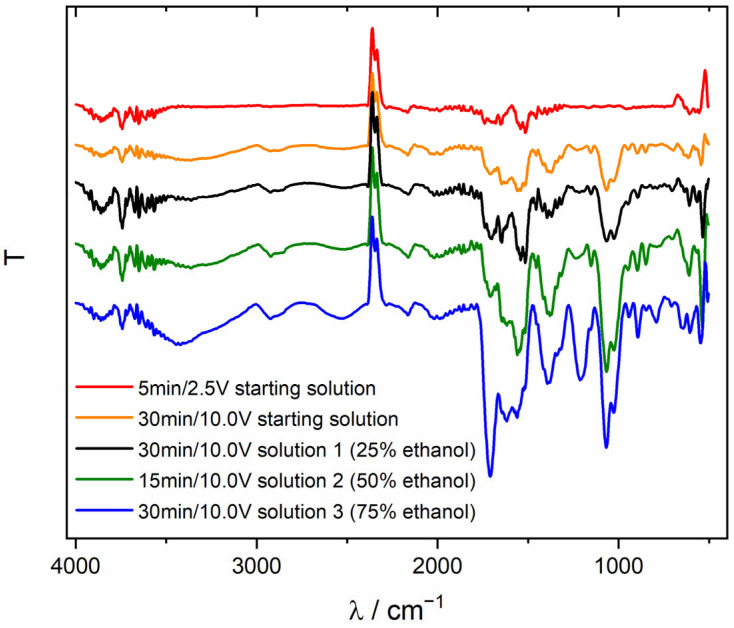
ATR-FTIR spectrum of the chitosan coatings deposited on the Ti substrate.

ATR-FTIR investigations identified characteristic functional groups within the chitosan structure (Figure 9). The peaks observed at 2924 cm^−1^, 1456 cm^−1^, and 1317 cm^−1^ correspond to the stretching vibrations of the methylene group (-CH_2_), commonly found in saccharide pyranose rings. The band observed at 1646 cm^−1^ is associated with the stretching vibrations of the C=O bond within the amide group. The peak at 1560 cm^−1^ comes from N-H bending vibrations. The 1377 cm^−1^ band resulted from symmetric vibrations of the CH_3_ groups. The bands at 1153 cm^−1^ and 894 cm^−1^ attest to the presence of a C-O-C bond. The absorption bands at 1068 cm^−1^ and 1024 cm^−1^ correspond to C-O stretching vibrations. In the ATR-FTIR spectra for a sample deposited for 30 min at 10 V from solution 3 (75% ethanol), an additional broad band of ~3356 cm^−1^ attributed to the O-H group is observed, which may indicate the presence of water molecules in the spatial network of chitosan [25,32,33,34,35].

### 3.3. Mechanical Study

**Figure 10:**
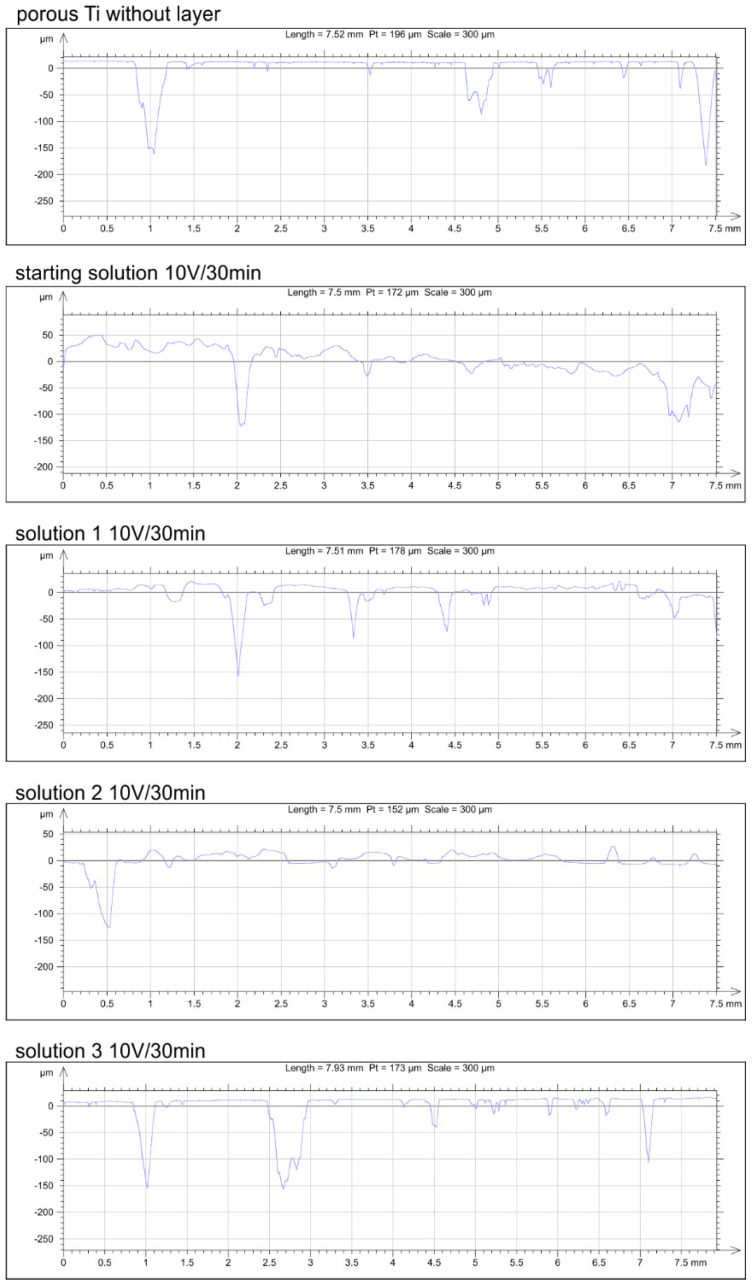
The roughness profile of porous Ti and chitosan coatings obtained at 10 V for 30 min from different solutions.

The effect of ethanol content in the deposition solution on the roughness of the chitosan coating was determined and summarized in Table 1 and Figure 10. The analysis compared the results for coatings deposited under the same process parameters: 10 V and 30 min. Based on the obtained results, it can be concluded that the coatings deposited from the starting solution and solution 1 exhibit the highest roughness, with similar arithmetical mean deviation, Ra, values of 3.32 μm. The lowest roughness was observed for coatings deposited from solutions 2 and 3, with the roughness profile being similar to that of the clean titanium substrate. The difference in roughness may be attributed to the amount of deposited polymer and the number of bubbles formed during the deposition process due to the release of hydrogen at the electrode. Moreover, the highest surface roughness could enhance the tissue integration and initial stabilization of the implant [36].

**Figure 11:**
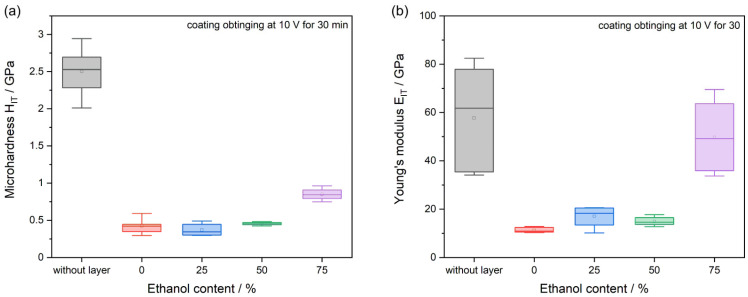
Chitosan coatings microhardness (**a**) and Young’s modulus (**b**) vs. ethanol content in deposition solution.

Figure 11 presents the dependence of the microhardness and Young’s modulus of the chitosan coating on the ethanol content in the deposition solution. The analysis compares samples that were deposited under identical conditions of 10 V for 30 min.

Based on the obtained results, there is an observable trend in increasing both microhardness and Young’s modulus for coatings deposited from solutions with higher ethanol concentrations. A significant increase in these values is particularly evident for the coating deposited from a solution containing 75% ethanol. The increased coating microhardness and stiffness may be associated with increased brittleness and susceptibility to cracking, as observed in the SEM images.

### 3.4. Biological Study

**Figure 12:**
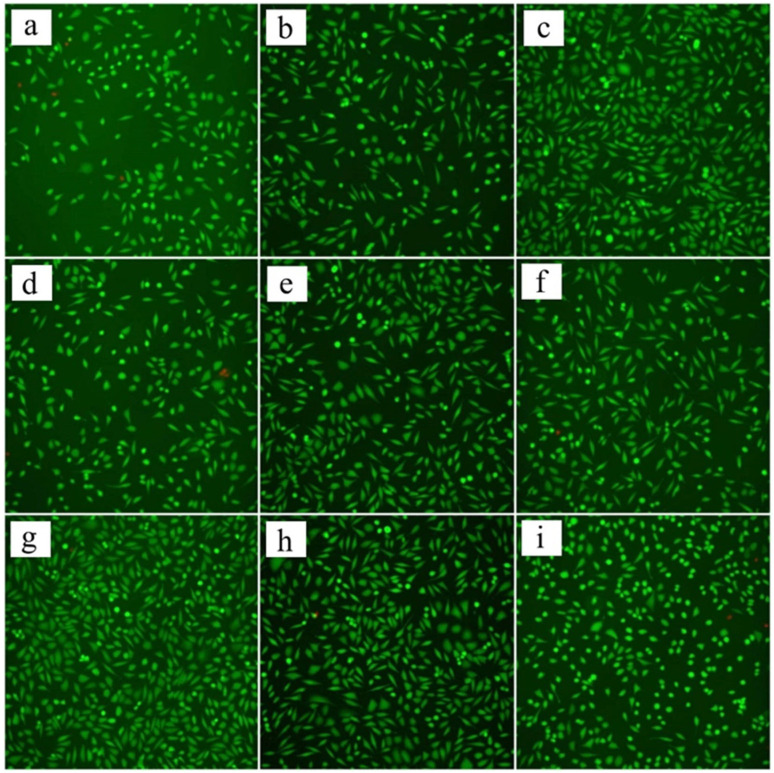
Cell viability: for control sample images (**a**–**c**), for samples deposited from solution 1 (2% citric acid solution in 25% ethanol containing 1 g/dm^3^ of chitosan) at 10 V for 30 min: images (**d**–**f**), for samples deposited from starting solution (2% citric acid solution containing 1 g/dm^3^ of chitosan) at 10 V for 30 min: images (**g**–**i**).

The results of the cytotoxicity assay study, presented in Table 2, show very high cell survival rates. The coatings deposited from the chitosan starting solution and the solution with ethanol addition are similar to the control sample. Based on the results, it could be concluded that the tested materials have no cytotoxic effect on fibroblasts. Figure 12 shows the results obtained in the cytotoxicity test. Few necrotic cells are visible for the control sample and the chitosan-coated materials, which can be observed because of their red coloration. The remaining cells show green fluorescence and indicate the presence of living cells.

Table 3 summarizes data on cell metabolic activity. The values for the control sample and the other tested samples with applied chitosan coatings, both in solution with and without ethanol, differ by small values. This indicates good metabolic activity of the cells in contact with the obtained biomaterials. Statistical analysis was carried out on this basis. The Shapiro–Wilk test showed that the distribution of the data is normal. This made it possible to carry out further statistical analysis (Table 4). The one-way ANOVA test results allow us to conclude that there were no statistically significant differences between the values of reassuring consumption in the cells of the control sample and the samples with the chitosan coating applied (Table 4). In the Levene test performed, no significant statistical differences were found between the variance values of the control sample and those with biopolymer coatings applied (Table 4).

After statistical tests, there is no cytotoxic effect of the produced chitosan coatings due to the lack of significant impact of its presence on the metabolic activity of cells. The result was also confirmed by vital staining, which showed a high cell survival rate of 99.71% for both study coatings.

## 4. Conclusions

1. The EPD method makes it possible to obtain chitosan-based biopolymer coatings on porous titanium substrates.
2. This study showed that this method allows the penetration of the coating material into the substrate’s pores.
3. It was also shown that it is possible to control the thickness and morphology of coatings by selecting deposition conditions. The deposition time and voltage increase, resulting in thicker coatings.
4. Moreover, the voltage increase in the electrophoretic deposition process promotes the formation of pores in chitosan.
5. The negative influence of ethanol content in the deposition solution was observed during this study. The increase in ethanol concentration increases the likelihood of cracking and peeling of the coating.
6. The deposition of coatings at 10 V for 30 min from the solutions resulted in coatings of good quality and effective coverage of the interior pores of the Ti substrate. Furthermore, these coatings exhibited the highest roughness, which may enhance the integration of the implant with the surrounding tissue.
7. Vital staining showed a high cell survival rate of 99.71%. The conducted tests show the absence of a cytotoxic effect of the produced biomaterial on fibroblast cells.

## Figures and Tables

**Table 1:** The surface roughness parameters of the chitosan coatings on porous Ti substrate.

	Without Layer	Starting Solution	Solution 1	Solution 2	Solution 3
Ra/μm	2.83	3.32	3.32	1.51	2.06
Rp/μm	5.64	7.26	6.67	3.16	4.41
Rz/μm	16.36	15.93	17.07	6.72	11.77

**Table 2:** Cell viability in the cytotoxicity assay.

Control Test	Starting Solution 2% Citric Acid Solution Containing 1 g/dm^3^ of Chitosan (30 min/10 V)	Solution 1 2% Citric Acid Solution in 25% Ethanol Containing 1 g/dm^3^ of Chitosan (30 min/10 V)
99.80%	99.71%	99.71%

**Table 3:** Metabolic activity expressed as a percentage of used substrate.

	**Control Sample**	Sample 1 (Solution 1 *) **30 min/10 V**	Sample 2 (Solution 1 *) **30 min/10 V**	Sample 1 (Starting Solution **) **30 min/10 V**	Sample 2 (Starting Solution **) **30 min/10 V**
	70.90%	65.79%	68.56%	63.73%	65.79%
	71.25%	68.35%	70.45%	68.75%	66.52%
	68.54%	65.89%	67.20%	64.89%	67.12%
	64.03%	59.78%	62.21%	54.89%	60.11%
	65.11%	60.42%	63.62%	59.26%	63.99%
Average	68%	64%	66%	62%	65%
STD	3%	4%	3%	5%	3%

* 2% citric acid solution in 25% ethanol containing 1 g/dm^3^ of chitosan, ** 2% citric acid solution containing 1 g/dm^3^ of chitosan.

**Table 4:** Statistical test results for samples deposited from starting solution and solution 1 at 30 min/10 V.

One-way ANOVA	F	d_f1_	d_f2_	P
	2.28	2	22	0.126
No sufficiently strong evidence exists to reject the null hypothesis of equality of mean values between groups.				
Levene test	F	d_f1_	d_f2_	P
	0.123	2	22	0.885
No sufficiently strong evidence exists to reject the null hypothesis of equality of variance values between groups.				
Shapiro–Wilk test	W		P	
	0.938		0.131	
No sufficiently strong evidence exists to reject the null hypothesis of normality of the data distribution.				

## Data Availability

The original contributions presented in the study are included in the article, further inquiries can be directed to the corresponding authors.
